# The effects of Covid-19 on Medical Education

**DOI:** 10.12669/pjms.38.1.5269

**Published:** 2022

**Authors:** Zoya Karim, Afzal Javed, Muhammad Waqar Azeem

**Affiliations:** 1 Dr. Zoya Karim, Medical Student, The University of Liverpool, Liverpool, L69 3BX, UK; 2 Dr. Afzal Javed, M.Phil; FRCPsych. Honorary Professor, Institute of Applied Health Research, University of Birmingham, UK. Chairman, Pakistan Psychiatric Research Centre, Fountain House, Lahore, Pakistan; 3 Prof. Muhammad Waqar Azeem, MD, DFAACAP, DFAPA. Chair, Department of Psychiatry, Sidra Medicine, Professor of Psychiatry, Weill Cornell Medical College, Director, World Psychiatric Association (WPA) Collaborating Center, Doha, Qatar

**Keywords:** Online education, e Learning, Covid-19 Pandemic

## Abstract

Since the start of January 2019, COVID-19 has caused unparalleled disruption to all aspects of life globally, including the delivery of medical education. Each university and institution have a different approach on how medicine, as a course, is taught and delivered, however, generally there is a consensus that in the earlier years, more emphasis should be given to the acquisition of theoretical and scientific knowledge, including anatomy and physiology. In the later years, medical students are then expected to apply their theoretical knowledge in a practical setting by attending various placements and interacting with patients and other healthcare professionals. The duality of this approach results in doctors who are both competent in their knowledge of the basic sciences whilst being good clinicians with sound practical and inter-personal skills. Covid-19 causes an unprecedented interruption to all students, however with courses such as Medicine, whereby a practical element is crucial, the adaptation to deliver the course effectively is more of a challenge. Clinical rotations were cancelled during the start of the pandemic due to concerns about the students and their families contracting the virus and also for the protection of the vulnerable patients in hospitals. In this manuscript we have explored the adaptations made in order to deliver medical education and evaluate the effectiveness of these methods. We will also be discussing the implications and limitations of these methods.

## ONLINE TEACHING

Due to progress, development and availability of technology, using online platforms to deliver lectures and workshops to medical students has been a mainstay of medical education throughout the pandemic. Online distance education can be delivered in one of two ways, those being asynchronous teaching and synchronous teaching. Asynchronous teaching involves pre-recorded lectures or podcasts that are accessible to students at their leisure and allow flexibility in the way they wish to learn. Synchronous teaching involves live sessions which encourage interaction between teachers and students. Studies have suggested that there is greater student satisfaction in using online distance learning and a blend of synchronous and asynchronous learning styles as it allows students to have more flexibility and control over their schedules and study at a time that is best suited to them.^1^ With the development of platforms such as Zoom and Microsoft Teams, virtual classrooms can be created giving students a chance to interact with other students and teachers alike and the use of “break-out rooms”, can be used to break down the classroom into even smaller groups allowing for discussion between small groups of students. This practically mimics a classroom scenario and skills such as communication and history-taking, which are pivotal in medicine can be taught and practiced. Whilst these skills are very important, it is hard to mimic physical examinations and procedural skills through these platforms. For example, it may be easy to teach and go through a cardiovascular examination with students through these platforms, however they must be practiced on other peers and eventually on patients in order to build confidence and in order to recognise certain signs. It is all well and good to describe what a collapsing pulse, for example, feels like however, a student is more likely to remember and recognize it in the future if they have felt it previously.^2^ Similarly, bedside manners are very difficult to develop if there is no or limited patient contact as this comes from being comfortable in a clinical setting and practicing inter-personal skills. For these reasons, medical students who are later on in their medical training, or those who are in their clinical years may be more disadvantaged by online distance learning than those who are in early stage of their training, which is purely theoretical and clinical exposure is not imperative.

The entire premise of delivering online learning is based on the assumption that all students will have a device from which they are able to view the content, as well as a stable internet connection and potentially access to a camera and microphone. Although, for most people, a smart device and internet connection is available, it is not possible to assume that across the globe, all students will have access to these facilities at a given time.^3^ Those who do not have access to these resources, will naturally be put at a disadvantage as facilities such as libraries and internet café’s have been closed due to the pandemic.

A study by Mukhtar et al has referred to the advantages of online learning which includes remote learning, comfort, and accessibility while the limitations of online learning were mentioned as inefficiency and difficulty in maintaining academic integrity.^4^ Another study by Abbasi et al has reported that students did not prefer e-teaching over face-to face teaching during the lockdown due to Covid pandemic.^5^

## IMPACT ON EXAMINATIONS

Due to the nature of the virus, most medical examinations were suspended due to the fact that students were not able to be in close proximity with each other and so alternative examination techniques had to be employed. Again, different universities used different approaches in order to examine their students, some opted for open-book examinations whilst others used online exam papers with invigilators monitoring the students to ensure cheating was not possible. Most medical schools in the UK also have practical exams towards the end of the academic year to ensure competency in practical skills such as history taking, examinations and procedural skills however these too, had to be cancelled.^6^ Often, patients who had agreed to help the medical students were brought in to the practical examinations and students would be expected to perform a thorough history and do a physical exam on the patient however due to the vulnerability of the patients and the close contact required this was no longer possible. Some universities opted for a simulated patient scenario whereby an actor was posing as a patient and students had to take a history however the physical examination element was not possible and therefore compromised. Using online examinations comes with its own merits as many final year students may not have been able to take their exams and therefore would not have been able to graduate and transition into doctors had online examinations not been an option. This could have been detrimental to both students and also to the general population as during the pandemic, as maximizing the number of doctors available would be of utmost importance. Again, the use of online platforms would rely on the fact that students had a stable internet connection and camera to ensure proper invigilation if that was the preferred examination technique. Online examinations also assume that students have an environment that is appropriate to take an examination. Some students may not have circumstances that are conducive to take an examination at home due to various reasons such as space, family reasons or other factors.

A study from Pakistan by Mumtaz et al has also highlighted the fact that lockdown has led in significant distortion in the academic word, significant disruption in internal assessment and examinations but it is poised to develop reliable, cost effective and secure online academic system..^7^

Another study from Pakistan which looked at the viewpoint of the medical and dental graduates on online learning during the Covid19 pandemic has reported that they welcomed the inclusion of e-learning into current teaching modalities. Though online learning may not provide flexibility in learning process but it saves time. Female students in particular showed more positive attitude towards e learning as compared to male students. Students also found easier access to online teaching resources. However, they were of the view that frequent participation in this learning process was important for the success of online education.^8^

## MENTAL HEALTH AND WELL-BEING OF STUDENTS

Undoubtedly, the mental health of the population, as well as medical students has been effected by the restrictions imposed due to COVID-19. Utmost efforts have been made by medical schools in order to ensure that medical education of a good standard is being imparted to medical students. However, personal circumstances and well-being also have an impact on how well the students are receiving the teaching. Firstly, with online teaching, asynchronous especially, students must find a way to make their own timetable, which in the midst of great uncertainty could be difficult. With day to day university life, there is a structure however achieving such structure without the support of formal teaching and your peers could prove to be difficult. It requires a level of motivation which may not have been present in some students due to the changing circumstances. Similarly, those who were expected to undertake examinations online during the pandemic would require some level of motivation in order to learn the content and study effectively. Trying to stick to a timetable and motivate one’s self through the lockdown restrictions of the pandemic could not have been an easy task, especially seeing as the uncertainty of the restrictions and the spread of the virus made it difficult to pin-point and end for normality to resume.

Another reason that may have instilled some anxiety in some medical students, especially those later on in their courses, is the fact they have done most of their learning for that year remotely and so having patient contact and performing practical skills in a clinical setting may be very daunting when it is time for them to resume their clinical placements. It may lead to a sense of incompetency just due to the fact that there has been limited exposure due to limited opportunities. However, when clinical rotations do resume, students most likely will adjust and acclimatize quickly.

In conclusion, the use of online learning platforms has allowed medical education and examinations to be delivered to students allowing them to progress with their degrees and minimize disruption, however this relies on the students’ individual circumstances alongside their ability to access a device with stable internet connection. The use of asynchronous and synchronous teaching could very well be integrated in future medical teaching (alongside formal teaching) as it allows for more flexibility and has been received well by students however solely depending on distance learning may create problems.

### Authors’ Contribution:

**ZK:** Conceived, writing & editing of manuscript. **AJ and MWZ:** Editing of manuscript.
